# Multiple sclerosis: 2024 update

**DOI:** 10.17879/freeneuropathology-2025-6762

**Published:** 2025-07-08

**Authors:** Luisa Klotz, Maija Saraste, Laura Airas, Tanja Kuhlmann

**Affiliations:** 1 Department of Neurology, University Hospital Münster, Münster, Germany; 2 Turku PET Centre, Turku University Hospital, Turku, Finland; 3 Neurocenter, Turku University Hospital, Turku, Finland; 4 Clinical Neurosciences, University of Turku, Turku, Finland; 5 InFLAMES Research Flagship, University of Turku, Turku, Finland; 6 Institute of Neuropathology, University Hospital Münster, Münster, Germany

**Keywords:** Multiple sclerosis, Genetics, Disease course, Diagnostic criteria, Biomarkers, Imaging, Treatment

## Abstract

Multiple sclerosis (MS) is a complex immune-mediated disease that leads to neurological disability, with ongoing challenges in understanding its initiation, predicting progression, and optimizing personalized treatment. This review article summarizes key research findings from 2024, covering advances in diagnostic criteria, understanding of pathophysiology, and treatment strategies. New studies reinforce the strong link between Epstein-Barr virus (EBV) and MS, while recent data point towards a role of genetics in MS disease progression. The 2024 McDonald criteria revision enhances diagnostic specificity and includes novel MRI markers and facilitates measurement of cerebrospinal fluid biomarkers. Additionally, recent genetic discoveries, advanced imaging techniques, and emerging biomarkers are refining disease monitoring and prognosis. Finally, we highlight promising therapeutic developments, including Bruton Tyrosine Kinase (BTK) inhibitors and CAR T-cell therapies, with the former representing a paradigm shift in the potential of targeting MS progression beyond focal inflammation.

## Introduction

Multiple sclerosis (MS) is a complex immune-mediated disease of the central nervous system and a leading cause of permanent neurological disability in young adults. Despite progress in reducing focal inflammatory activity through high-efficacy immunomodulatory treatments, major challenges remain in MS research. Our incomplete understanding of disease initiation in susceptible individuals hinders the development of preventive treatments. Additionally, the variability in disease course makes it difficult to predict outcomes and individualize therapy for optimal efficacy and minimal side effects ("treat-to-target"). Effective monitoring of this heterogeneous disease and its treatment requires a personalized approach using reliable imaging and biomarkers that offer insight into individual disease biology. Furthermore, recent discoveries on myeloid and B cells in disease progression have driven the development of novel therapies.

**Figure 1:**
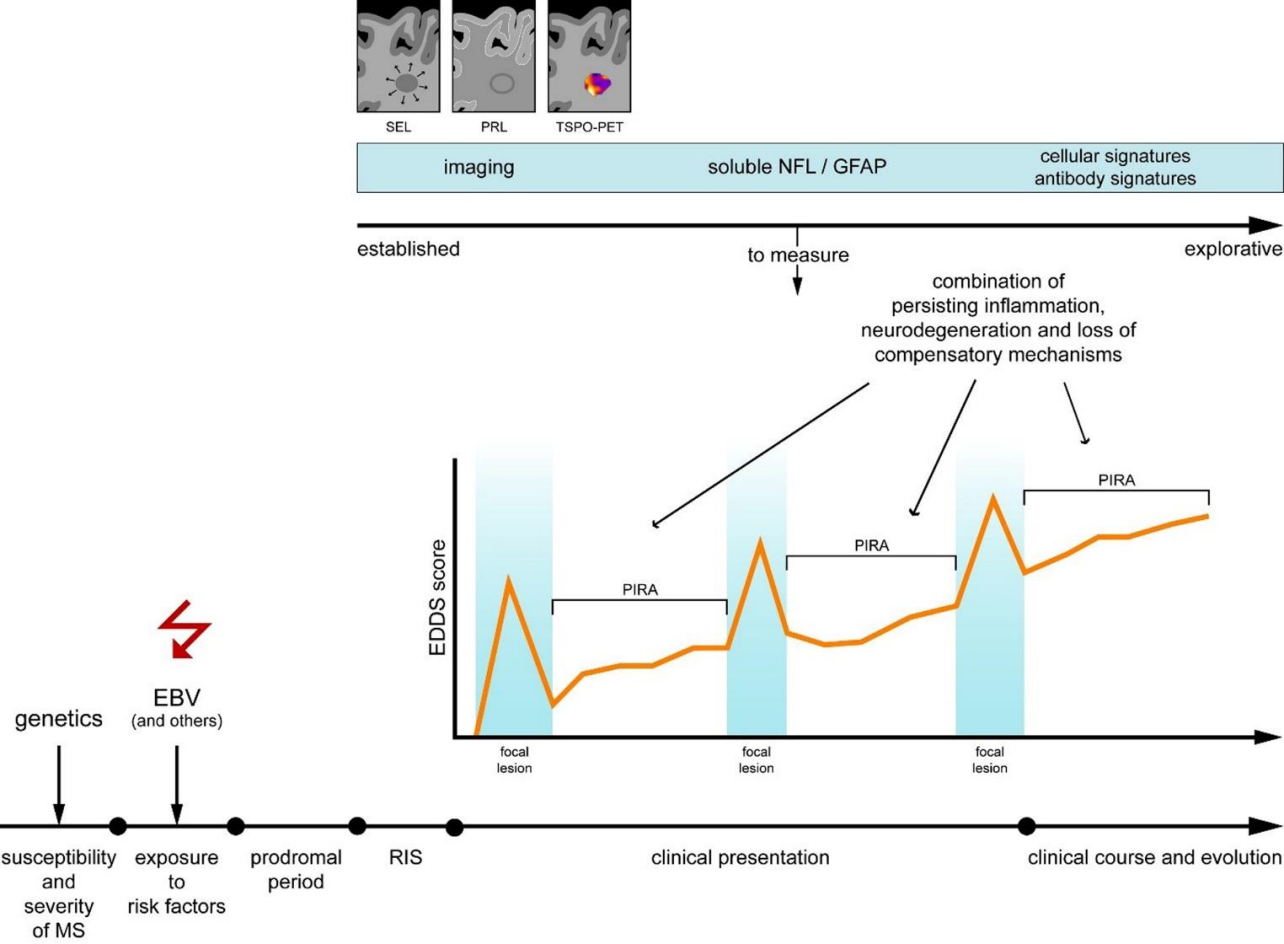
Schematic illustration depicting the MS disease course and established and explorative tools to diagnose MS and monitor or predict the disease course.

 Based on these considerations, we have selected publications from 2024 that provide new insights into MS pathophysiology, evolving diagnostics, novel biomarkers, imaging techniques, and emerging treatment strategies (**Fig. 1**). Monozygotic twin studies revealed a heritability risk of approximately 25–30 %. So far, studies have identified up to 233 genetic variants linked to MS susceptibility, most expressed in immune cells^1^. In 2023, the first study focused on MS severity and identified one significant risk allele and 11 suggestive loci, all encoding for genes expressed in the CNS^2^. Further 2024 research has explored correlations between these SNPs and aspects of disease severity. 

 A role of EBV in MS pathogenesis has long been suspected and several studies provide compelling evidence supporting this connection. Nearly all MS patients have prior EBV infection, and a landmark study showed EBV increases MS risk more than 30-fold^3^. In 2024, research confirmed that pediatric-onset MS is strongly associated with EBV, differentiating it from MOG antibody-associated disease (MOGAD). MS patients exhibit dysregulated immune responses to EBV, suggesting impaired control of latent infection, raising the potential for antiviral therapies. 

Accurate, early MS diagnosis remains crucial for improved long-term treatment outcomes. The 2024 McDonald criteria revision aims at enhancing specificity while maintaining sensitivity. Key updates include recognizing optic nerve involvement as part of dissemination in space (DIS), incorporating MRI markers like the central vein sign (CVS) and paramagnetic rim lesions (PRL), and using kappa-free light chains (kFLC) as an alternative to oligoclonal bands (OCBs) in cerebrospinal fluid analysis. Furthermore, accumulating evidence indicates that MS pathology begins long before clinical onset. Consequently, radiologically isolated syndrome (RIS), identified via incidental MRI findings, is now recognized as a preclinical MS stage allowing MS diagnosis even in the absence of clinical manifestation, with disease-modifying therapies shown to delay conversion to MS. We also highlight recent findings on the MS prodromal stage, characterized by early nonspecific symptoms such as fatigue, depression, and sleep disturbances.

Biomarkers are essential for individual disease prognosis and prediction but remain underutilized in MS. Serum neurofilament light (NfL) is the most advanced biomarker, correlating with disease activity and long-term disability risk. Glial fibrillary acidic protein (GFAP) has emerged as a complementary biomarker, and findings from 2024 suggest their combination might help to distinguish inflammatory damage from neurodegeneration. Furthermore, promising new data point towards novel approaches to improve patient stratification and predict treatment responses.

Novel imaging markers like CVS and PRL enhance specificity of MS diagnosis, and in particular PRLs are considered as novel biomarker of disease progression. Besides novel imaging approaches, the implementation of AI-driven analysis of imaging and clinical data facilitates both diagnosis and prognosis, potentially enabling earlier intervention and personalized therapy in the future.

Recent findings challenge the traditional view that MS disability is primarily relapse-driven, as progression independent of relapse activity (PIRA) significantly contributes to long-term disability, even in early MS. Reflecting this conceptual shift, we discuss recent clinical trial outcomes on Bruton Tyrosine kinase (Btk) inhibitors as the most appreciated clinical highlight in MS research in 2024. Furthermore, we discuss novel cellular therapies such as CAR T cells, which target deep tissue B-cell depletion, offering a new frontier in MS treatment in the future.

## Pathogenesis of MS

### MS and genetics

 Based on family and twin studies, the heritability of MS is estimated to be approximately 30 %^4^. HLA-DRB1*15:01 is the strongest MS risk factor, which increases the MS risk threefold in individuals carrying at least one copy of the allele^4^. Barrie and colleagues now demonstrated that the genetic risk of MS rose among the pastoralists in the Pontic steppe and was brought to Europe by the Yamnaya-related migration approximately 5000 years ago. The authors analyzed datasets from the Mesolithic, medieval and post-medieval periods and found a positive selection of MS associated immunogenetic variant risk genes. Interestingly, most of the alleles under positive selection were associated with protective effects against specific pathogens and/or infectious diseases, suggesting that transmission of pathogens drove the selection of immune gene variants, which are now associated with an increased risk of autoimmune diseases^4^. 

 In 2019, the International MS Genetic consortium provided a detailed genetic and genomic map of multiple sclerosis^1^. This study identified 200 autosomal susceptibility variants outside the major histocompatibility complex (MHC), one chromosome X variant, and 32 within the extended MHC. These genes associated with MS susceptibility are implicated in multiple innate and adaptive pathways (e.g. TNFa, and type 1 interferons) and cells of the immune system including microglia, thus strongly supporting the immune-driven nature of disease onset. The authors of these studies estimated that their results could explain 48 % of MS heritability. However, only in 2023, the first SNP associated with disease severity (reflected by the age-related MS severity score) was identified in a genome-wide association study including data from 12,584 cases and replicated in an additional study comprising further 9,805 cases^2^. The authors found a significant association with rs10191329 in the *DYSF–ZNF638 *locus. DYSF is well known for its function in muscle membrane repair and calcium dependent membrane fusion, whereas ZNF638 is a transcriptional co-activator, which is involved in cell differentiation and proliferation^5^. Both genes are expressed by neurons and glia cells, suggesting that disease severity may be influenced by Central Nervous System (CNS) intrinsic mechanisms. The authors could also show that rs10191329 was linked to higher lesion load in brain stem and cortex in a large MS autopsy cohort, however the molecular pathways and relevant cell types mediating this phenotype are unknown^2^. Maybe as important than identifying gene variants associated with MS severity, the authors observed a significant heritability enrichment in CNS tissue, further suggesting that CNS resident cells determine severity and outcome of the disease^2^. 

 Interestingly, in 2024, an independent imaging study including a discovery cohort of 748 and a replication study of 360 people with relapsing remitting MS observed an association with 28 % more brain atrophy per rs10191329*A allele, further corroborating its role in pathophysiological processes underlying disease progression. The authors therefore encourage stratification for rs10191329 in clinical trials^6^. In contrast, other studies were not able to detect a correlation between rs10191329 and longitudinal binary disease severity or other clinically relevant outcomes^7,8^. However, the sample size of the aforementioned studies was modest compared to the initial study and the estimated effect size of rs10191329 rather low, further strengthen the necessity for collaborative research approaches to maximize sample sizes to further disentangle the genetic background of MS. 

### MS and EBV

 EBV has been implicated in the pathogenesis of MS for a long time as reviewed and summarized in a number of well-written and informative reviews^9,10^. Approximately 90 % of the population are infected by EBV within the first two decades in life; the virus is transmitted by saliva or infectious B cells. After infection, the virus establishes latency resulting in its lifelong persistence. Primary infection during childhood is usually asymptomatic, but the majority of individuals infected during adolescence or adulthood will develop infectious mononucleosis. A seminal longitudinal study published in *Science* 2022 studying a cohort of more than 10 million young adults on active duty in the US military, among them 955 who were diagnosed with MS during their period of service, demonstrated that EBV infection increases the risk to develop MS more than 30-fold^3^. In contrast, previous studies reported lower rates of EBV infection among children with pediatric onset of MS raising questions about whether EBV infection is indeed pre-requisite across the age spectrum^11^. However, those studies were conducted before MOG antibody tests were broadly available and therefore these studies could not differentiate reliably between pediatric onset-MS and MOG antibody-associated disease (MOGAD). In this line, a recent study differentiating between MOGAD and pediatric onset MS now demonstrated that 96 % of the children with pediatric onset MS had antibodies directed against the viral capsid of EBV and 90 % had antibodies directed against EBNA1 (a marker of a remote EBV infection) further supporting the notion that EBV infection is required to trigger MS across the whole age spectrum^12^. Interestingly, children with MOGAD had similar rates of EBV seropositivity as healthy children, indicating that EBV infection is not a risk factor for MOGAD. 

 The mechanisms underlying the increased MS risk associated with EBV infection are still poorly understood. Results from previous studies suggest that the EBV infection is less well controlled in people with MS^13,14^. This concept is further supported by a number of findings published during the last year: 1.) The EBV antibody response is not limited to EBNA1, suggesting a larger dysregulation of EBV-specific antibody responses than previously recognized in pwMS^15^. 2.) PwMS, but not individuals with other neuroinflammatory diseases including neuromyelitis optica, MOGAD or Susac’s syndrome display an aberrant MHC-I-restricted T cell response directed against EBV^16^ and 3.) the frequencies of CXCR3+ memory B cells are reduced in the blood of genetically identical twins with MS compared to their unaffected siblings. Based on the latter finding, the authors propose that these memory B cells migrate into the CNS, mature into antibody secreting cells in the CNS and drive the disease^17^. 4.) Spontaneous lymphoblastoid cell lines (SLCLs) isolated from pwMS with active disease had higher EBV lytic gene expression than SLCLs from MS patients with stable disease or HCs. Furthermore, LCLs from patients with active disease displayed activation of selected inflammatory pathways and of genes associated with the lytic gene expression of EBV indicating that dysregulation of EBV gene expression by B cells drives a pro-inflammatory, pathogenic B cell phenotype^18^. Interestingly, the authors provide also evidence that antiviral approaches targeting EBV replication decreased cytokine production and autologous CD4+ T cell responses. The identification of EBV as an important contributing factor of MS raises the questions whether anti-viral treatment approaches could either prevent or slow down MS disease progression and first clinical trials are currently under way^10,19^. 

### Novel insight into MS pathophysiology using new technologies

 There are ongoing efforts to characterize molecular signatures associated with MS lesion types, remyelination failure or neurodegeneration using modern sequencing technologies, such as sc/snRNA sequencing or spatial transcriptomics (ST) to identify new pharmacological treatment targets^20^. Spatial transcriptomics enables spatially resolved analysis of gene expression within intact tissue sections in contrast to bulk or single-cell RNA sequencing (scRNA-seq), which requires the dissociation of tissues and loses spatial context. Lerma-Martin and colleagues analyzed 12 subcortical MS lesions from six donors and seven controls by combining spatial transcriptomics (10x Genomics Visium Spatial Gene Expression platform) with snRNA sequencing. The comparison between histologically annotated areas and unsupervised molecularly defined niches revealed a significant overlap both at the cluster and tissue section level validating the reliability of the method^20^. Alemsa and colleagues performed a similar study using two different spatial transcriptomic platforms (10x Genomics Visium Spatial Gene Expression platform, Nanostring GeoMX )^21^. Both publications provide further insights into potential cell-cell communication via receptor ligand interactions, which will contribute to the disentanglement of the dynamic cellular and molecular changes occurring in MS lesions. Furthermore, they compared the transcriptional profiles in perilesional WM and NAWM and observed lesion-type dependent alterations, further supporting the notion that perilesional white matter directly adjacent to a lesion differs from NAWM, which is in line with observations from histopathology and imaging^23^. Together, these data suggest, that the inflammatory infiltrates in active and mixed active/inactive lesions affect the perilesional tissue environment. However, although spatial transcriptomics is a valuable new tool for the identification of disease associated molecular pathways in MS, the techniques used in the above mentioned manuscript do not reach single cell resolution. A new method, named in situ sequencing allows the identification of transcriptomic patterns with single cell resolution. Kukanja and colleagues used this method to disentangle molecular mechanisms underlying lesion formation in experimental autoimmune encephalitis, an animal model of MS^26^. They also identified disease-associated glia cells, which were detected outside of EAE lesions, and which were dynamically induced and resolved during the EAE course in line with the observation of disease associated changes also outside of MS lesions. The authors could also provide evidence that the method is suitable to analyze human MS tissue samples. A limitation of these techniques are the limited number of genes that can be analyzed (in this study 239 and 266 genes in mouse and human samples, respectively); however there are ongoing efforts to enable whole transcriptome single cell scale analysis in in formalin fixed paraffin embedded tissue sections^27^. 

### Preclinical stages of MS – expanding the MS disease continuume

 Historically, MS diagnosis required a clinically defined event, but recent evidence indicates that disease processes begin well before symptoms emerge. MRI studies have shown that asymptomatic individuals can have lesions suggestive of inflammatory demyelination, with some later developing clinical MS – a condition termed radiologically isolated syndrome (RIS), first described by Okuda et al. in 2009^28^. RIS is a condition in which asymptomatic individuals exhibit MRI lesions in characteristic locations that are highly suggestive for MS. In 2023, the RIS Consortium refined the diagnostic criteria for RIS: individuals with lesions in at least three key CNS locations (periventricular, juxtacortical, infratentorial, or spinal cord) fulfill RIS imaging criteria.^29^ Alternatively, those having lesions in only one or two of these areas but additionally exhibiting with two of the following criteria – spinal cord lesion, CSF-restricted oligoclonal bands, or new demyelinating lesions on follow-up MRI – fulfill the RIS definition. This revision improves prognostic stratification, as studies report conversion rates to clinical MS of 34 % at five years and 51 % at ten years. Additional risk factors for conversion into MS include younger age (< 37 years), elevated IgG index, detection of more than two CSF-restricted OCBs, the presence of infratentorial or spinal cord lesions, as well as contrast-enhancing lesions on a follow-up MRI. Notably, recent clinical trials performed in RIS have demonstrated that oral disease-modifying treatments such as dimethyl fumarate and teriflunomide can delay or prevent conversion to clinical MS, supporting the concept that effective intervention is possible prior to clinical onset.^30^ Based on these considerations, some RIS constellations now fulfill the new diagnostic criteria of MS, which are discussed below, which illustrates that biological rather than purely clinical considerations are now implemented in our current concept of MS diagnosis. 

 Beyond imaging, a range of nonspecific clinical symptoms – including depression, anxiety, fatigue, sleep disturbances, and headache – have been identified as part of the so-called MS prodrome^31^. Recently, studies in both children and adults confirm that these early signs, along with elevated serum neurofilament light chain levels up to nine years before clinical onset, support the concept that MS pathophysiology initiates long before overt clinical symptoms^32^. However, due to the unspecific nature of these symptoms and a high overlap with other immune-mediated diseases, it is currently not possible to provide a clear MS prodrome definition that may guide further diagnostic workup to facilitate early MS diagnosis. 

## New diagnostic framework of MS

### Diagnosis of MS – the 2024 update of the McDonald criteria

 Diagnosing multiple sclerosis requires balancing early detection with minimizing misdiagnosis. Over two decades, diagnostic criteria have evolved with MRI and CSF biomarkers, enabling earlier and more accurate diagnosis. After the last revision of diagnostic criteria in 2017^35^, another update has been proposed in 2024. This update is based on novel data highlighting the role of the optic nerve, the relevance of RIS, the necessity to differentiate MS from other autoimmune conditions like NMOSD and MOGAD, acknowledgement of the concept that disease progression is a key feature of relapsing MS, and finally, the diagnostic challenge of MS in older individuals and those with comorbidities. Based on these emerging concepts, the following changes have been presented at the ECTRIMS congress in 2024 by X. Montalban on behalf of the International Advisory Committee on Clinical Trials in MS^36,37^, however the diagnostic criteria have not been published yet. First, the optic nerve is now included as a diagnostic region providing evidence for the dissemination in space (DIS), and besides clinical manifestation as optic neuritis, optic nerve involvement can be illustrated by optical coherence tomography, visual evoked potential and MRI. Second, MS diagnosis requires documentation of DIS in at least two of five CNS regions (optic nerve, cortical/juxtacortical, periventricular, infratentorial, spinal cord), if this is supplemented with either dissemination in time, or detection of oligoclonal bands or kappa free light chains in the CSF. Notably, the dissemination in time (DIT) is no longer mandatory for diagnosis, as this is not exclusive to MS and imaging variability can affect its interpretation. With regard to imaging, two new imaging criteria have newly been implemented that are not mandatory but may facilitate diagnosis based on their high specificity for MS: The central vein sign (CVS), caused by inflammatory lesions forming around central veins, can be visualized by T2* MR imaging, and detection of at least 6 CVS can confirm MS in a situation when only two topographies are affected^38^. Paramagnetic rim lesions (PRL) indicate chronic active MS lesions and can be detected by susceptibility-weighted MRI sequences, which are sensitive for detection of iron accumulation in a rim of myeloid cells around chronic MS lesions^38^. These PRLs are not exclusively found in MS but display high specificity; therefore, detection of at least one PRL in the presence of either DIT or CSF positivity can now confirm MS in cases with so far only one topography affected. 

 For CSF analysis in the context of MS workup, the kappa-free light chain (kFLC) index can now replace OCB detection, both reflecting intrathecal immunoglobulin production^39^. This may facilitate diagnostic procedures as this is a cost-effective and rater-independent method based on nephelometry or turbidimetry. Furthermore, as described in the previous section, evidence supports RIS as part of the MS continuum, with over half of RIS cases developing clinical MS within 10 years^40^. Therefore, the diagnostic criteria now allow for MS diagnosis in those RIS patients exhibiting lesions in at least two topographies plus DIT or CSF positivity. This represents a real conceptual change as this accepts MS as a biological diagnosis even in the absence of any clinical manifestation. Regarding the former separation of diagnostic criteria for relapsing versus progressive MS, the common biological mechanisms of these disease courses have been acknowledged, and therefore, the newly revised diagnostic criteria can be applied to both disease courses. Finally, criteria have been adapted in individuals over 50 or with comorbidities acknowledging the increased risk of MS misdiagnosis in these conditions due to small vessel disease, migraine or other inflammatory disorders, which can also present with T2 lesions. In these cases, additional criteria should be fulfilled, such as detection of at least one spinal cord lesion, positive CSF and/or detection of CVS. Together, this 2024 revision of the MS diagnostic criteria aims to facilitate the diagnosis of MS in individuals based on biological considerations without compromising specificity, which will ultimately improve clinical outcomes of people with MS globally. 

## Monitoring of disease activity

### Biomarkers and their use for personalized medicine – where do we stand?

Biomarkers are key to advancing personalized medicine by enabling precise diagnosis, risk stratification, and guidance in treatment responses. In oncology, the use of biomarkers for personalized medicine approaches is already firmly established due to the unique genetic and molecular profiles of a patient’s tumor. In autoimmune diseases, personalized medicine based on biomarkers is still in its infancy, primarily due to the complexity of their pathophysiology and the heterogeneity in clinical presentation.

 In the field of MS, recent publications illustrate both advances and limitations in this area of research. Besides MRI, serum neurofilament light chain (NfL) levels represent the most advanced biomarker for facilitating the assessment of a patient’s individual prognosis^41^. Although NfL is a non-specific marker of neuronal injury and therefore not limited to MS, elevated serum NfL (sNfL) levels have been associated with acute relapse activity and responses to highly active treatments^42^. Recently, the relevance of NfL in predicting disability accumulation has been further explored, demonstrating that elevated sNfL levels after a first demyelinating event are associated with an increased risk of future disability accumulation^43^. 

 Furthermore, glial fibrillary acidic protein (GFAP) has emerged as another serum biomarker in MS. One study proposed that the combined assessment of GFAP and NfL may help distinguish between acute focal inflammatory damage – reflected by NfL elevation – and relapse-independent progression, as indicated by GFAP elevation^44^. In contrast, a more recent study by Monreal and colleagues suggested that higher NfL levels were associated with an increased risk of both relapse-associated worsening (RAW) and PIRA, confirming its prognostic value at disease onset. Higher GFAP levels were linked only to a higher risk of reaching an EDSS score of 3, but not to RAW or PIRA. However, in a subset of patients with low NfL levels, GFAP was also associated with PIRA. In individuals with low levels of both markers, the risk for all outcomes was lowest. These findings suggest that GFAP may indicate progression only in a subset of patients, potentially driven by a distinct pathophysiology, and that combining both markers may enhance prediction accuracy^45^. 

 Additional experimental approaches have been published, highlighting the potential of more pathophysiology-driven biomarkers to improve patient stratification in MS. One study, based on the concept that MS has a strong genetic component, used a genetic risk score (GRS) in individuals with optic neuritis (ON) to predict the future development of MS^46^. The study demonstrated that combining genetic data with demographic factors significantly improved the prediction of MS in individuals with undifferentiated ON, with results replicated in an independent cohort. Another study analyzed whole-proteome autoantibody profiles in individuals both before and after MS onset. Notably, around 10 % of MS patients displayed a unique signature that could already be detected several years before disease onset^47^. In line with other evidence pointing toward the relevance of certain virus-immune interactions as prerequisites for MS development in susceptible individuals, this antibody signature included a common motif observed in several human infectious pathogens, including EBV. Although present in only a smaller subset of MS patients, these findings raise the possibility that at least a fraction of individuals at high risk of developing MS could be identified before clinical disease onset, potentially facilitating the implementation of preventive strategies in the future. 

 A German study employed high-dimensional immunological profiling of peripheral blood in untreated early MS patients to identify subgroups based on distinct immunological characteristics^47^. This approach enabled the identification of three distinct subgroups with unique immune patterns – one associated with high inflammatory activity based on clinical and imaging measures, and another characterized by early signs of tissue destruction and neurodegeneration. Notably, these subgroups not only exhibited differences in clinical disease trajectories but also in response to immune treatments, highlighting that a better characterization of distinct immunobiological patterns may help predict individual treatment responses in the future. Similarly, analyzing the so far largest MS brain tissue collection comprising normal appearing white matter as well as grey and white matter lesions revealed different cellular compositions between the lesions but surprisingly similar cell-type gene expression patterns both within and across patients, suggesting patient-dependent global changes. Based on these observations, the authors stratified the patients into different molecular subgroups suggesting that different molecular mechanisms may drive pathophysiology and predict response to treatments targeting CNS intrinsic disease mechanism. However, the correlation of these molecular subtypes with pathological or clinical disease trajectories has not yet been established^22^. 

### Novel advances in imaging to detect disease progression

 Conventional MRI detects focal lesions with great sensitivity, but it does not perform well in detecting the diffuse pathology responsible for PIRA. The imminent need of treatments for slowing down disease progression in MS has prompted wide interest in the application of advanced imaging methods to better understand and assess the progression-promoting pathological processes within the CNS. A number of comprehensive review articles on imaging of focal and diffuse compartmentalized inflammatory and neurodegenerative processes were published in 2024^48^. Potential imaging biomarkers of MS progression include detection of paramagnetic lesions (PRLs) using iron-sensitive MRI sequences, identification of cortical lesions using double inversion recovery (DIR), assessment of grey matter damage, and measurement of choroid plexus volume. 

**Figure 2: Histological and imaging detection of chronic active lesions.**
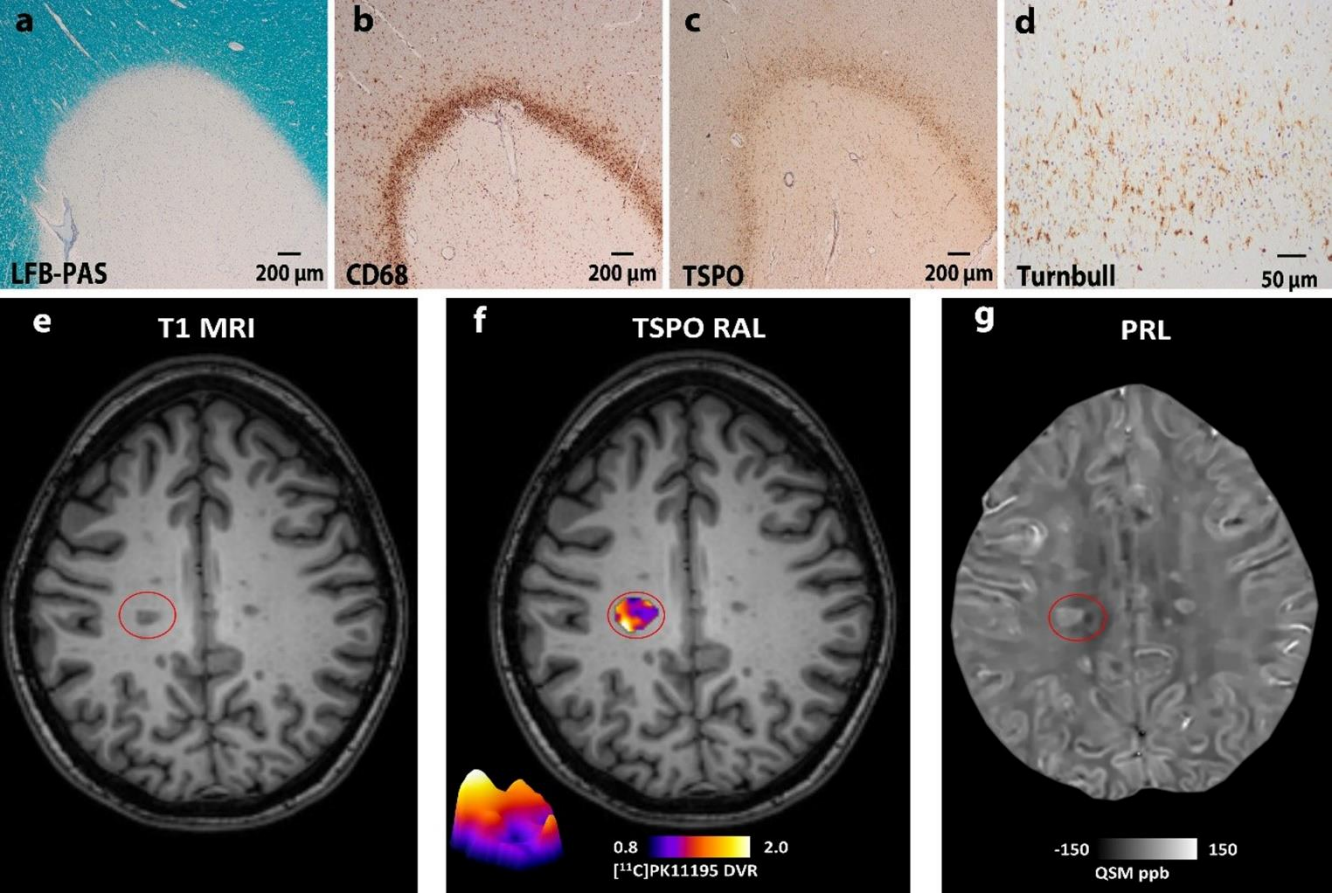
Histological characterization of a chronic active lesions (= mixed active/inactive lesions) (**a to d**). The lesion is completely demyelinated and has a sharp border to the adjacent normal appearing white matter (Luxol-fast blue staining) (**a**). Chronic active lesions are characterized by a rim of myeloid cells expressing CD68 and TSPO (**b and c**). A subset of chronic active lesions displays a dense rim of myeloid cells with cytoplasmic iron depositions (Turnbull staining) (**d**). Different imaging techniques, such as TSPO-PET and quantitative susceptibility mapping (QSM) are currently used to identify this lesion type in individuals with MS (**e to g**). The red circle in indicates a hypointense T1 lesions (**e**), that displays a TSPO positive rim in TSPO PET (RAL: rim active lesion) (**f**) and a paramagnetic rim in QSM (PRL: paramagnetic rim lesion) (**g**).

 In advanced clinical imaging, PRLs, slowly expanding lesions (SELs) and TSPO-rim-active lesions are considered to represent chronic active lesions (CALs) (also termed mixed active/inactive lesions)^50^, which are characterized by a hypocellular lesion center and a rim of macrophages/microglia (**Fig. 2**). However, which of these different methods is the best predictor of disease progression has yet to be determined. Notably, recent work suggests that there is only partial overlap in CAL-detection using these imaging methods. Here, numbers of SELs were shown to be higher than those of PRLs (616 vs 80), and the correlation between lesion counts was quite moderate (ρ = 0.28, *p* = 0.03)^54^. This suggests that SEL and PRL may capture distinct pathophysiological features of chronic active lesions. Similarly, based on a recent publication, TSPO-PET has a higher sensitivity to detect more CALs compared to susceptibility weighted MRI, although there was a correlation between the number of ^11^C-PBR28 active lesions and PRLs in 7T phase images^55^. Moreover, this study found that TSPO-PET whole active lesion volume had the strongest association with the EDSS score in a cohort of 30 study patients including equal numbers of patients with RRMS and SPMS^55^. PRLs have been particularly widely studied during the past years and are now considered a predictive imaging biomarker for greater disease severity and progression and correlates with brain and spinal cord atrophy^53^. A consensus statement developed by the North American Imaging in Multiple Sclerosis (NAIMS) Cooperative published in 2024 provides guidance for the definition and measurement of PRLs to promote their clinical translation^51^. This also prompted their inclusion in the new diagnostic criteria for MS as described above. 

 Following this line, several articles published in 2024 provided further evidence regarding the association of PRLs with disease progression: .1) Patients with PIRA had significantly more PRLs, also when analysis was restricted to patients with RRMS^56^. 2) PRLs associated with PIRA over the 2 years after study entry demonstrating their predictive power for PIRA ^38^. 3) A longitudinal study with median follow-up time of 5.6 years showed that the appearance of new PRLs was associated with increased rates of PIRA^57^. On the other hand, PRL disappearance was associated with reduced rates of confirmed disability progression, suggesting the need of additional studies to understand the predictive value of these dynamic changes. 

 Imaging tools to assess neurodegeneration are another approach to predict disease progression. Higher baseline cortical lesion load, but not white matter lesion load or new cortical lesions during the observation period of three years, predicted disability worsening suggesting that cortical lesions forming earlier during the disease course contribute to disability progression rather than new cortical lesions^58^. However, an alternative explanation could be that the detrimental effects of cortical lesions on disease progression require time to manifest clinically. Thalamic atrophy is well-known to be associated with disease progression. The study by Cagol and colleagues now provides further insight into the underlying mechanisms of thalamic atrophy. Using advanced quantitative MRI, they demonstrated that microstructural thalamic changes linked to demyelination, neuroaxonal loss, and disturbances in iron homeostasis correlated well with clinical disability, cognitive impairment, and MRI measures of disease burden^59^. Interestingly, a recent longitudinal study provided further evidence about the gradual increase of choroid plexus volume (1.4 % per year) and its association with brain atrophy^60^. In this study from Sydney, 57 patients with RRMS underwent annual MRI scans during a minimum follow-up of four years. Interestingly, they showed that the annual change in choroid plexus volume correlated with chronic lesion expansion (r = 0.46, p < 0.001), further supporting the notion that plexus enlargement is at least partly induced by cellular or molecular inflammatory mediators. An association between choroid plexus volume, cognitive impairment, and fatigue was also recently demonstrated^61^. 

 There are also several other advanced imaging methods that have shown promise for identification of disease progression, such as the diffusion MRI based neurite orientation dispersion and density imaging (NODDI) that provides specific measures of tissue microstructure, soma and neurite density imaging (SANDI), and leptomeningeal enhancement that can be visualized using delayed post-contrast FLAIR, demonstrating meningeal B cell aggregates^53^. 

### Use of AI

 The potential of artificial intelligence (AI) and machine learning continues to be explored in the context of MS as reviewed by Collorone et al^62^. AI has been used in explorative studies to guide MS diagnosis, prediction, lesion segmentation, and investigation of disease mechanisms. The authors conclude that although there are several challenges regarding the quality of input data and ethical issues, the use of AI has made significant progress in recent years in the MS field. However, the reproducibility and validation of the results, which is important for the integration of AI based methods into clinical practice, warrants further studies. 

 In 2024, Noteboom et al^63^ studied how various machine learning models were capable of determining clinical impairment at baseline and of predicting future clinical worsening in two cohorts (n = 123 and 330, respectively). Support vector machine classifier was the best AI tool to identify higher disability (EDSS ≥ 4) and impaired cognition (SDMT Z-score ≤ −1.5) when clinical factors and global or regional MRI volumes were used as input. However, the machine learning models were not able to predict clinical worsening after two or five years. On the other hand, Andorra et al showed that Random Forest algorithms predicted NEDA (no evidence of disease activity) with AUC 0.80 and confirmed disability accumulation with AUCs 0.62, 0,63 and 0.61 for EDSS, SDMT and 9HPT, respectively^64^. Algorithms were first tested using a prospective multi-centric cohort including 322 patients with MS and 98 healthy controls, and then using a prospective cohort of 271 patients with MS. Their findings suggest, that combining clinical, and imaging and in some instances also omics data with machine learning may help identifying MS patients at risk of disability worsening. 

 Proper identification of focal MS lesions is the basis for successful diagnosis, treatment and disease monitoring. Furthermore, reliable identification of chronic active lesions gives valuable information on the risk of later progression as described above. Manual lesion segmentation is time-consuming, but thus far it has proven more reliable than existing automated lesion segmentation methods. This is now being challenged by recent lesion-detection work using deep learning models. Pasquale De Rosa et al applied a consensus-based framework, which combines five publicly available deep learning models, to improve lesion segmentation^65^. They used two datasets, including 131 and 30 patients with MS to compare it with lesions masks segmented manually. Their method showed good agreement with the volume and numbers of lesions identified through manual segmentation (ρ = 0.92 and ρ = 0.97, and ρ = 0.83 and ρ = 0.94 for datasets I and II, respectively). Automated methods for PRL assessment are also urgently needed. Now the NAIMS Cooperative have tested an Automated Paramagnetic Rim Lesion (APRL) algorithm in a multi-center setting and reported that this automated segmentation method successfully captured 115 (78 %) of manually identified PRLs^66^. This gives promise for facilitated PRL-detection in large datasets; an improvement that could enable routine PRL detection in clinical settings and in the context of treatment trials. 

## Rethinking MS Progression and Treatment Strategies

### The concept of progression independent from relapse activity and emerging treatments targeting the pathophysiology of progression

 While it has long been accepted that disability accrual in MS is primarily driven by focal inflammation as the pathophysiological correlate of relapses, recent research has identified significant disease progression independent of relapse activity, termed PIRA. Identification of PIRA became possible through careful re-evaluation of clinical trial data from high-efficacy treatments – particularly ocrelizumab – where progression was observed despite an almost complete absence of relapses^67^. In 2023, data from a large CIS / early MS cohort revealed that PIRA can occur even in early relapsing MS and is associated with an unfavorable long-term prognosis^68^. Although several studies have shown that current treatments partially influence PIRA^69^, none of the approved therapies are sufficient to control it, and its exact pathophysiology remains elusive, hampering the development of targeted treatments. 

 In this context, a novel class of drugs – Bruton’s tyrosine kinase (BTK) inhibitors – has gained attention in MS treatment. BTK inhibitors block BTK, an enzyme essential for intracellular signaling during B cell receptor activation and for activating myeloid cells, without directly affecting T cells^70^. Several BTK inhibitors have already been approved for hematologic malignancies such as chronic lymphocytic leukemia and mantle cell lymphoma; their dual action on B cells and myeloid cells has sparked interest for autoimmune diseases^70^. BTK inhibition modulates both adaptive and innate immune responses by suppressing proinflammatory cytokine production, antigen presentation, and cell survival^71^. The recognized role of B cells in MS pathophysiology, along with the contribution of myeloid cells to chronic lesion formation and progression, has led to multiple trials in both relapsing and progressive MS^70^. Data from several recent trials investigating evobrutinib and tolebrutinib have been presented in 2024. Notably, evobrutinib failed to demonstrate superiority over teriflunomide (which interferes with lymphocyte proliferation and metabolic activity) in controlling focal inflammatory activity and confirmed accumulation of disability^72^, whereas tolebrutinib showed efficacy in reducing confirmed disability progression in three clinical trials studying its efficacy in people with RRMS and SPMS (GEMINI I and II and HERCULES trials)^73,74^. Recently, new data have been presented demonstrating that the effect of tolebrutinib on disability progression was only seen in patients with PRLs at baseline^75^. This is particularly remarkable as tolebrutinib exerted only modest effects on markers of acute focal inflammation, therefore providing first evidence that progression-related pathology can be modulated independently of acute focal activity and this is centered around modulation of B cells and/or myeloid cells^73,74^. 

### Emerging cellular therapies – CAR T cells

 In other autoimmune diseases, novel cellular therapies are increasingly being explored for their potential applications. One of the most promising candidates already in clinical use in oncology are chimeric antigen receptor (CAR) T cells^76^. These are T cells that have been genetically modified to express a construct enabling high-affinity antigen recognition and downstream signaling, thereby conferring proliferative capacity, effector function, and persistence in the recipient for a durable therapeutic effect^77^. Importantly, the CAR T receptor is not restricted by MHC for antigen recognition. In most cases, this is done autologously – T cells are obtained from the patient, engineered *ex vivo* with CAR constructs, expanded, and then reinfused. The concept currently under evaluation in neuroimmunology, including MS, involves CAR T cells targeting B cells (via CD19 or BCMA) to achieve thorough B cell depletion even in tissues, as these cells have demonstrated good deep tissue penetration, including the CNS^78,79^. Indeed, several case series have recently been published illustrating profound effects of this therapeutic approach in several patients with refractory myasthenia gravis and stiff person syndrome, however results from clinical trials are not yet available^78^^,80^^,81^. 

 Our current understanding of MS progression centers on a compartmentalized inflammatory process that limits the efficacy of primarily peripherally acting DMTs and CAR T cells may represent an emerging technology to tackle this treatment challenge. Published 2024 data from two progressive MS patients treated with a single dose of fully humanized second-generation CD19 CAR T cells showed persistence of CAR T cells in both the peripheral blood and CSF^82^. Notably, CAR T cell treatment resulted in peripheral B cell depletion and a sustained reduction of CSF oligoclonal bands in one patient, providing indirect evidence of effective CNS plasma cell depletion. Although this small case series with a limited observation time precludes definitive efficacy evaluation, the treatment was well tolerated, with only mild cytokine release syndrome and no ICANS. Several clinical trials in relapsing and progressive MS are underway to further explore this promising approach and assess potential side effects, including secondary malignancies, which would strongly limit use of this approach in the context of autoimmunity. 

### Preclinical studies

 The search for neuroprotective and remyelination promoting therapies continues, but is hampered by the limited understanding of the molecular and cellular mechanisms driving neurodegeneration and remyelination failure in MS. Oligodendrocytes and neurons are not passive targets of the immune response, as extensive previous research suggest that oligodendrocytes as well as neurons can launch an inflammatory response^83^. The group led by Manuel Friese discovered, in detailed and elaborate in vitro and in vivo studies, the stimulator of interferon genes (STING) as an important regulator in neurons, which is essential for the homeostasis of the neuronal red-ox system and inflammation-induced ferroptosis. STING is upregulated in neurons in EAE and in people with MS and most interesting both, pharmacological and genetic ablation of STING protected against inflammation-induced neurodegeneration in EAE mice. Sting1-cKO EAE mice displayed ameliorated EAE disease course and increased numbers of surviving neurons, but no differences in the extent of inflammatory infiltrates suggesting that pharmacological targeting of STING may represent a direct neuroprotective treatment approach^88^. 

 Another focus in preclinical MS research is to unravel the underlying molecular mechanism for remyelination failure. Current concepts suggest that not only a differentiation block of OPC into mature myelinating oligodendrocytes but also an impaired myelin sheath formation by mature oligodendrocytes may contribute to remyelination failure in MS^89^. A potential explanation for the latter might be that mature oligodendrocytes are epigenetically silenced, as suggested by the work of Liu and colleagues^90^. They screened an epigenetic compound library and identified the small molecule ESI1 that promoted the maturation and/or in vitro myelination of primary mouse and human iPSC derived oligodendrocytes, promoted remyelination in different animal models and improved clinical signs in the EAE model. In summary, this study identifies epigenetic silencing of mature oligodendrocytes as a new pathomechanism contributing to remyelination failure in MS and provides evidence that targeting the epigenetic machinery might be a promising new pharmacological target to overcome remyelination failure in MS. 

## Summary

Conceptually, the identification of the first SNP associated with disease severity represents a major breakthrough, since it suggests a genetic component in the pathophysiology of disease progression independent from the immune system. The accumulating evidence of EBV as important trigger of MS disease onset raises the fascinating possibility of an anti-viral treatment which may either stop or prevent MS. The new diagnostic framework of MS establishes MS as a disease continuum and allows diagnosis based on purely biologic considerations in the absence of any clinical manifestation and new imaging and biomarkers as well as AI approaches may facilitate the prediction of the disease course and treatment responses in individual pwMS in the not too far future.

A conceptual game changer from a therapeutic perspective are the results from the tolebrutinib study program, as they demonstrate for the first time that tackling clinical outcomes of disease progression is feasible beyond targeting focal inflammation. The ultimate challenge for the future will be the translation of our continuously increasing understanding of MS pathophysiology into new treatment approaches to successfully stop or prevent MS.

## Conflict of interest statement

L.K. receives research support from the German Research Foundation (DFG), the Interdisciplinary Center for Clinical Research (IZKF) Münster, National MS Society, Biogen, Novartis and Merck Serono. She received compensation for serving on scientific advisory boards and speaker honoraria from Alexion, Amgen, Argenx, Bayer, Biogen, Bristol-Myers Squibb, Grifols, Hexal, Horizon, Janssen, Merck Serono, Novartis, Roche, Sandoz, Sanofi, Santhera, Teva and Viatris.

L.A. receives grants from the Research Council of Finland, Aatos Erkko Foundation, US National MS Society, MerckSerono and Sanofi. She received speaker and advising honoraria from Sanofi, Biogen, Novartis, Kiniksa, Continuum Therapeutics and Merck.

M.S. declares no conflicts of interest.

T.K. receives research funding from the German Research Foundation, Interdisciplinary Center for Clinical Research (IZKF) Münster, National MS Society, German MS Society and Novartis. She received compensation for serving on scientific advisory boards from Novartis, Sanofi and Merck and speaker honoraria from Novartis, Biogen, Sanofi and Roche.

## References

[R1] International Multiple Sclerosis Genetics, C. Multiple sclerosis genomic map implicates peripheral immune cells and microglia in susceptibility. Science 365(2019). 10.1126/science.aav7188 PMC7241648 31604244

[R2] International Multiple Sclerosis Genetics, C. & Multiple, M.S.C. Locus for severity implicates CNS resilience in progression of multiple sclerosis. Nature 619, 323-331 (2023). 10.1038/s41586-023-06250-x PMC10602210 37380766

[R3] Bjornevik, K., et al. Longitudinal analysis reveals high prevalence of Epstein-Barr virus associated with multiple sclerosis. Science 375, 296-301 (2022). 10.1126/science.abj8222 35025605

[R4] Barrie, W., et al. Elevated genetic risk for multiple sclerosis emerged in steppe pastoralist populations. Nature 625, 321-328 (2024). 10.1038/s41586-023-06618-z PMC10781639 38200296

[R5] Meruvu, S., Hugendubler, L. & Mueller, E. Regulation of adipocyte differentiation by the zinc finger protein ZNF638. J Biol Chem 286, 26516-26523 (2011). 10.1074/jbc.M110.212506 PMC3143616 21602272

[R6] Gasperi, C., et al. A Genetic Risk Variant for Multiple Sclerosis Severity is Associated with Brain Atrophy. Ann Neurol 94, 1080-1085 (2023). 10.1002/ana.26807 PMC11303986 37753809

[R7] Campagna, M.P., et al. No evidence for association between rs10191329 severity locus and longitudinal disease severity in 1813 relapse-onset multiple sclerosis patients from the MSBase registry. Mult Scler 30, 1216-1220 (2024). 10.1177/13524585241240406 PMC11363458 38511853

[R8] Kreft, K.L., et al. Relevance of Multiple Sclerosis Severity Genotype in Predicting Disease Course: A Real-World Cohort. Ann Neurol 95, 459-470 (2024). 10.1002/ana.26831 37974536

[R9] Bjornevik, K., Munz, C., Cohen, J.I. & Ascherio, A. Epstein-Barr virus as a leading cause of multiple sclerosis: mechanisms and implications. Nat Rev Neurol 19, 160-171 (2023). 10.1038/s41582-023-00775-5 36759741

[R10] Giovannoni, G. Targeting Epstein-Barr virus in multiple sclerosis: when and how? Curr Opin Neurol 37, 228-236 (2024). 10.1097/WCO.0000000000001266 38511407

[R11] Banwell, B., et al. Clinical, environmental, and genetic determinants of multiple sclerosis in children with acute demyelination: a prospective national cohort study. Lancet Neurol 10, 436-445 (2011). 10.1016/S1474-4422(11)70045-X 21459044

[R12] Fadda, G., et al. Epstein-Barr Virus Strongly Associates With Pediatric Multiple Sclerosis, But Not Myelin Oligodendrocyte Glycoprotein-Antibody-Associated Disease. Ann Neurol 95, 700-705 (2024). 10.1002/ana.26890 38411340

[R13] Munger, K.L., Levin, L.I., O'Reilly, E.J., Falk, K.I. & Ascherio, A. Anti-Epstein-Barr virus antibodies as serological markers of multiple sclerosis: a prospective study among United States military personnel. Mult Scler 17, 1185-1193 (2011). 10.1177/1352458511408991 PMC3179777 21685232

[R14] DeLorenze, G.N., et al. Epstein-Barr virus and multiple sclerosis: evidence of association from a prospective study with long-term follow-up. Arch Neurol 63, 839-844 (2006). 10.1001/archneur.63.6.noc50328 16606758

[R15] Thomas, O.G., et al. Heightened Epstein-Barr virus immunity and potential cross-reactivities in multiple sclerosis. PLoS Pathog 20, e1012177 (2024). 10.1371/journal.ppat.1012177 PMC11156336 38843296

[R16] Schneider-Hohendorf, T., et al. Broader anti-EBV TCR repertoire in multiple sclerosis: disease specificity and treatment modulation. Brain 148, 933-940 (2025). 10.1093/brain/awae244 PMC11884754 39021292

[R17] Ingelfinger, F., et al. Twin study dissects CXCR3(+) memory B cells as non-heritable feature in multiple sclerosis. Med 5, 368-373 e363 (2024). 10.1016/j.medj.2024.02.013 PMC11018360 38531361

[R18] Soldan, S.S., et al. Multiple sclerosis patient-derived spontaneous B cells have distinct EBV and host gene expression profiles in active disease. Nat Microbiol 9, 1540-1554 (2024). 10.1038/s41564-024-01699-6 PMC11900839 38806670

[R19] van de Waterweg Berends, A., Broux, B., Machiels, B., Gillet, L. & Hellings, N. The EBV-MS connection: the enigma remains. Front Immunol 15, 1466339 (2024). 10.3389/fimmu.2024.1466339 PMC11390381 39267757

[R20] Lerma-Martin, C., et al. Cell type mapping reveals tissue niches and interactions in subcortical multiple sclerosis lesions. Nat Neurosci 27, 2354-2365 (2024). 10.1038/s41593-024-01796-z PMC11614744 39501036

[R21] Alsema, A.M., et al. Spatially resolved gene signatures of white matter lesion progression in multiple sclerosis. Nat Neurosci (2024). 10.1038/s41593-024-01765-6 39501035

[R22] Macnair, W., et al. snRNA-seq stratifies multiple sclerosis patients into distinct white matter glial responses. Neuron 113, 396-410 e399 (2025). 10.1016/j.neuron.2024.11.016 39708806

[R23] Tonietto, M., et al. Periventricular remyelination failure in multiple sclerosis: a substrate for neurodegeneration. Brain 146, 182-194 (2023). 10.1093/brain/awac334 36097347

[R24] Kessler, W., Thomas, C. & Kuhlmann, T. Microglia activation in periplaque white matter in multiple sclerosis depends on age and lesion type, but does not correlate with oligodendroglial loss. Acta Neuropathol 146, 817-828 (2023). 10.1007/s00401-023-02645-2 PMC10628007 37897549

[R25] Bodini, B., et al. Individual Mapping of Innate Immune Cell Activation Is a Candidate Marker of Patient-Specific Trajectories of Worsening Disability in Multiple Sclerosis. J Nucl Med 61, 1043-1049 (2020). 10.2967/jnumed.119.231340 PMC7383077 32005777

[R26] Kukanja, P., et al. Cellular architecture of evolving neuroinflammatory lesions and multiple sclerosis pathology. Cell 187, 1990-2009 e1919 (2024). 10.1016/j.cell.2024.02.030 38513664

[R27] Oliveira, M.F., et al. Characterization of immune cell populations in the tumor microenvironment of colorectal cancer using high definition spatial profiling. bioRxiv, 2024.2006.2004.597233 (2024). 10.1101/2024.06.04.597233

[R28] Okuda, D.T., et al. Incidental MRI anomalies suggestive of multiple sclerosis: the radiologically isolated syndrome. Neurology 72, 800-805 (2009). 10.1212/01.wnl.0000335764.14513.1a 19073949

[R29] Lebrun-Frenay, C., et al. The radiologically isolated syndrome: revised diagnostic criteria. Brain 146, 3431-3443 (2023). 10.1093/brain/awad073 PMC11004931 36864688

[R30] Lebrun-Frenay, C., et al. Teriflunomide and Time to Clinical Multiple Sclerosis in Patients With Radiologically Isolated Syndrome: The TERIS Randomized Clinical Trial. JAMA Neurol 80, 1080-1088 (2023). 10.1001/jamaneurol.2023.2815 PMC10442780 37603328

[R31] Epstein, S.E. & Longbrake, E.E. Shifting our attention earlier in the multiple sclerosis disease course. Curr Opin Neurol 37, 212-219 (2024). 10.1097/WCO.0000000000001268 38546031

[R32] Britze, J., et al. Temporal Dynamics of Plasma Neurofilament Light in Blood Donors With Preclinical Multiple Sclerosis. Neurol Neuroimmunol Neuroinflamm 12, e200335 (2025). 10.1212/NXI.0000000000200335 PMC11616971 39602675

[R33] Guinebretiere, O., et al. Association Between Diseases and Symptoms Diagnosed in Primary Care and the Subsequent Specific Risk of Multiple Sclerosis. Neurology 101, e2497-e2508 (2023). 10.1212/WNL.0000000000207981 PMC10791050 38052493

[R34] Akmatov, M.K., et al. Symptoms Prior to Diagnosis of Multiple Sclerosis in Individuals Younger Than 18 Years. JAMA Netw Open 7, e2452652 (2024). 10.1001/jamanetworkopen.2024.52652 PMC11681376 39729316

[R35] Thompson, A.J., et al. Diagnosis of multiple sclerosis: 2017 revisions of the McDonald criteria. Lancet Neurol 17, 162-173 (2018). 10.1016/S1474-4422(17)30470-2 29275977

[R36] Montalban, X. ECTRIMS 2024 - Oral Presentations. Multiple Sclerosis Journal 30, 4-124 (2024). 10.1177/13524585241269218

[R37] Levraut, M., Landes-Chateau, C., Mondot, L., Cohen, M. & Lebrun-Frenay, C. The Kappa Free Light Chains Index and Central Vein Sign: Two New Biomarkers for Multiple Sclerosis Diagnosis. Neurol Ther 14, 711-731 (2025). 10.1007/s40120-025-00737-7 PMC12089642 40189723

[R38] Borrelli, S., et al. Central Vein Sign, Cortical Lesions, and Paramagnetic Rim Lesions for the Diagnostic and Prognostic Workup of Multiple Sclerosis. Neurol Neuroimmunol Neuroinflamm 11, e200253 (2024). 10.1212/NXI.0000000000200253 PMC11129678 38788180

[R39] Vecchio, D., et al. Kappa index for multiple sclerosis diagnosis: an accurate biomarker of intrathecal synthesis. J Neurol 272, 30 (2024). 10.1007/s00415-024-12826-y 39666131

[R40] Lebrun-Frenay, C., et al. Radiologically isolated syndrome. Lancet Neurol 22, 1075-1086 (2023). 10.1016/S1474-4422(23)00281-8 37839432

[R41] Bittner, S., Oh, J., Havrdova, E.K., Tintore, M. & Zipp, F. The potential of serum neurofilament as biomarker for multiple sclerosis. Brain 144, 2954-2963 (2021). 10.1093/brain/awab241 PMC8634125 34180982

[R42] Benkert, P., et al. Serum Glial Fibrillary Acidic Protein and Neurofilament Light Chain Levels Reflect Different Mechanisms of Disease Progression under B-Cell Depleting Treatment in Multiple Sclerosis. Ann Neurol 97, 104-115 (2024). 10.1002/ana.27096 PMC11683165 39411917

[R43] Monreal, E., et al. Association of Serum Neurofilament Light Chain Levels at Disease Onset With Disability Worsening in Patients With a First Demyelinating Multiple Sclerosis Event Not Treated With High-Efficacy Drugs. JAMA Neurol 80, 397-403 (2023). 10.1001/jamaneurol.2023.0010 PMC9972238 36848127

[R44] Meier, S., et al. Serum Glial Fibrillary Acidic Protein Compared With Neurofilament Light Chain as a Biomarker for Disease Progression in Multiple Sclerosis. JAMA Neurol 80, 287-297 (2023). 10.1001/jamaneurol.2022.5250 PMC10011932 36745446

[R45] Monreal, E., et al. Serum biomarkers at disease onset for personalized therapy in multiple sclerosis. Brain 147, 4084-4093 (2024). 10.1093/brain/awae260 39101570

[R46] Loginovic, P., et al. Applying a genetic risk score model to enhance prediction of future multiple sclerosis diagnosis at first presentation with optic neuritis. Nat Commun 15, 1415 (2024). 10.1038/s41467-024-44917-9 PMC10902342 38418465

[R47] Zamecnik, C.R., et al. An autoantibody signature predictive for multiple sclerosis. Nat Med 30, 1300-1308 (2024). 10.1038/s41591-024-02938-3 PMC11980355 38641750

[R48] Comi, G., et al. Assessing disease progression and treatment response in progressive multiple sclerosis. Nat Rev Neurol 20, 573-586 (2024). 10.1038/s41582-024-01006-1 39251843

[R49] Rocca, M.A., et al. Current and future role of MRI in the diagnosis and prognosis of multiple sclerosis. Lancet Reg Health Eur 44, 100978 (2024). 10.1016/j.lanepe.2024.100978 PMC11496980 39444702

[R50] Dal-Bianco, A., Oh, J., Sati, P. & Absinta, M. Chronic active lesions in multiple sclerosis: classification, terminology, and clinical significance. Ther Adv Neurol Disord 17, 17562864241306684 (2024). 10.1177/17562864241306684 PMC11660293 39711984

[R51] Bagnato, F., et al. Imaging chronic active lesions in multiple sclerosis: a consensus statement. Brain 147, 2913-2933 (2024). 10.1093/brain/awae013 PMC11370808 38226694

[R52] Scalfari, A., et al. Smouldering-Associated Worsening in Multiple Sclerosis: An International Consensus Statement on Definition, Biology, Clinical Implications, and Future Directions. Ann Neurol 96, 826-845 (2024). 10.1002/ana.27034 39051525

[R53] Calabrese, M., et al. Determinants and Biomarkers of Progression Independent of Relapses in Multiple Sclerosis. Ann Neurol 96, 1-20 (2024). 10.1002/ana.26913 38568026

[R54] Calvi, A., et al. Relationship between paramagnetic rim lesions and slowly expanding lesions in multiple sclerosis. Mult Scler 29, 352-362 (2023). 10.1177/13524585221141964 PMC9972234 36515487

[R55] Treaba, C.A., et al. Phenotyping in vivo chronic inflammation in multiple sclerosis by combined (11)C-PBR28 MR-PET and 7T susceptibility-weighted imaging. Mult Scler 30, 1755-1764 (2024). 10.1177/13524585241284157 PMC11742271 39436837

[R56] Cagol, A., et al. Association of Spinal Cord Atrophy and Brain Paramagnetic Rim Lesions With Progression Independent of Relapse Activity in People With MS. Neurology 102, e207768 (2024). 10.1212/WNL.0000000000207768 PMC10834139 38165377

[R57] Reeves, J.A., et al. Associations Between Paramagnetic Rim Lesion Evolution and Clinical and Radiologic Disease Progression in Persons With Multiple Sclerosis. Neurology 103, e210004 (2024). 10.1212/WNL.0000000000210004 PMC11509899 39447104

[R58] Beck, E.S., et al. Contribution of new and chronic cortical lesions to disability accrual in multiple sclerosis. Brain Commun 6, fcae158 (2024). 10.1093/braincomms/fcae158 PMC11137753 38818331

[R59] Cagol, A., et al. Advanced Quantitative MRI Unveils Microstructural Thalamic Changes Reflecting Disease Progression in Multiple Sclerosis. Neurol Neuroimmunol Neuroinflamm 11, e200299 (2024). 10.1212/NXI.0000000000200299 PMC11409727 39270143

[R60] Klistorner, S., et al. Longitudinal enlargement of choroid plexus is associated with chronic lesion expansion and neurodegeneration in RRMS patients. Mult Scler 30, 496-504 (2024). 10.1177/13524585241228423 PMC11010552 38318807

[R61] Preziosa, P., et al. Chronic Active Lesions and Larger Choroid Plexus Explain Cognition and Fatigue in Multiple Sclerosis. Neurol Neuroimmunol Neuroinflamm 11, e200205 (2024). 10.1212/NXI.0000000000200205 PMC11073888 38350048

[R62] Collorone, S., et al. Artificial intelligence applied to MRI data to tackle key challenges in multiple sclerosis. Mult Scler 30, 767-784 (2024). 10.1177/13524585241249422 38738527

[R63] Noteboom, S., et al. Evaluation of machine learning-based classification of clinical impairment and prediction of clinical worsening in multiple sclerosis. J Neurol 271, 5577-5589 (2024). 10.1007/s00415-024-12507-w PMC11319410 38909341

[R64] Andorra, M., et al. Predicting disease severity in multiple sclerosis using multimodal data and machine learning. J Neurol 271, 1133-1149 (2024). 10.1007/s00415-023-12132-z PMC10896787 38133801

[R65] De Rosa, A.P., et al. Consensus of algorithms for lesion segmentation in brain MRI studies of multiple sclerosis. Sci Rep 14, 21348 (2024). 10.1038/s41598-024-72649-9 PMC11393062 39266642

[R66] Chen, L., et al. Multicenter validation of automated detection of paramagnetic rim lesions on brain MRI in multiple sclerosis. J Neuroimaging 34, 750-757 (2024). 10.1111/jon.13242 PMC12370289 39410780

[R67] Kappos, L., et al. Contribution of Relapse-Independent Progression vs Relapse-Associated Worsening to Overall Confirmed Disability Accumulation in Typical Relapsing Multiple Sclerosis in a Pooled Analysis of 2 Randomized Clinical Trials. JAMA Neurol 77, 1132-1140 (2020). 10.1001/jamaneurol.2020.1568 PMC7281382 32511687

[R68] Tur, C., et al. Association of Early Progression Independent of Relapse Activity With Long-term Disability After a First Demyelinating Event in Multiple Sclerosis. JAMA Neurol (2022). 10.1001/jamaneurol.2022.4655 PMC9856884 36534392

[R69] Portaccio, E., et al. Progression is independent of relapse activity in early multiple sclerosis: a real-life cohort study. Brain 145, 2796-2805 (2022). 10.1093/brain/awac111 35325059

[R70] Kramer, J., Bar-Or, A., Turner, T.J. & Wiendl, H. Bruton tyrosine kinase inhibitors for multiple sclerosis. Nat Rev Neurol 19, 289-304 (2023). 10.1038/s41582-023-00800-7 PMC10100639 37055617

[R71] De Bondt, M., Renders, J., Struyf, S. & Hellings, N. Inhibitors of Bruton's tyrosine kinase as emerging therapeutic strategy in autoimmune diseases. Autoimmun Rev 23, 103532 (2024). 10.1016/j.autrev.2024.103532 38521213

[R72] Montalban, X., et al. Safety and efficacy of evobrutinib in relapsing multiple sclerosis (evolutionRMS1 and evolutionRMS2): two multicentre, randomised, double-blind, active-controlled, phase 3 trials. Lancet Neurol 23, 1119-1132 (2024). 10.1016/S1474-4422(24)00328-4 39307151

[R73] Fox, R.J., et al. Tolebrutinib in Nonrelapsing Secondary Progressive Multiple Sclerosis. N Engl J Med 392, 1883-1892 (2025). 10.1056/NEJMoa2415988 40202696

[R74] Oh, J., et al. Tolebrutinib versus Teriflunomide in Relapsing Multiple Sclerosis. N Engl J Med 392, 1893-1904 (2025). 10.1056/NEJMoa2415985 40202623

[R75] Oh, J., et al. Paramagnetic Rim Lesions as a Prognostic and Predictive Biomarker in the Tolebrutinib Phase 3 Trials for Disability Outcomes. ACTRIMS Forum 2025 - Platform and Invited. Multiple sclerosis Journal. 31, 3-23 (2025). 10.1177/1352458525133322

[R76] Mougiakakos, D., Meyer, E. & Schett, G. CAR T-cells in autoimmunity: game changer or stepping stone? Blood (2024).10.1182/blood.202402541339700499

[R77] Ransohoff, R.M. Selected Aspects of the Neuroimmunology of Cell Therapies for Neurologic Disease: Perspective. Neurol Neuroimmunol Neuroinflamm 12, e200352 (2025). 10.1212/NXI.0000000000200352 PMC11649171 39671535

[R78] Mougiakakos, D., et al. Successful generation of fully human, second generation, anti-CD19 CAR T cells for clinical use in patients with diverse autoimmune disorders. Cytotherapy 27, 236-246 (2025). 10.1016/j.jcyt.2024.09.008 39530971

[R79] Tur, C., et al. CD19-CAR T-cell therapy induces deep tissue depletion of B cells. Ann Rheum Dis 84, 106-114 (2025). 10.1136/ard-2024-226142 39874224

[R80] Faissner, S., et al. Successful use of anti-CD19 CAR T cells in severe treatment-refractory stiff-person syndrome. Proc Natl Acad Sci U S A 121, e2403227121 (2024). 10.1073/pnas.2403227121 PMC11214089 38885382

[R81] Haghikia, A., et al. Anti-CD19 CAR T cells for refractory myasthenia gravis. Lancet Neurol 22, 1104-1105 (2023). 10.1016/S1474-4422(23)00375-7 37977704

[R82] Fischbach, F., et al. CD19-targeted chimeric antigen receptor T cell therapy in two patients with multiple sclerosis. Med 5, 550-558 e552 (2024). 10.1016/j.medj.2024.03.002 38554710

[R83] Jakel, S., et al. Altered human oligodendrocyte heterogeneity in multiple sclerosis. Nature 566, 543-547 (2019). 10.1038/s41586-019-0903-2 PMC6544546 30747918

[R84] Kirby, L., et al. Oligodendrocyte precursor cells present antigen and are cytotoxic targets in inflammatory demyelination. Nat Commun 10, 3887 (2019). 10.1038/s41467-019-11638-3 PMC6715717 31467299

[R85] Di Liberto, G., et al. Neurons under T Cell Attack Coordinate Phagocyte-Mediated Synaptic Stripping. Cell 175, 458-471 e419 (2018). 10.1016/j.cell.2018.07.049 30173917

[R86] Andreadou, M., et al. IL-12 sensing in neurons induces neuroprotective CNS tissue adaptation and attenuates neuroinflammation in mice. Nat Neurosci 26, 1701-1712 (2023). 10.1038/s41593-023-01435-z PMC10545539 37749256

[R87] Alves de Lima, K., et al. Meningeal gammadelta T cells regulate anxiety-like behavior via IL-17a signaling in neurons. Nat Immunol 21, 1421-1429 (2020). 10.1038/s41590-020-0776-4 PMC8496952 32929273

[R88] Woo, M.S., et al. STING orchestrates the neuronal inflammatory stress response in multiple sclerosis. Cell 187, 4043-4060 e4030 (2024). 10.1016/j.cell.2024.05.031 38878778

[R89] Klotz, L., Antel, J. & Kuhlmann, T. Inflammation in multiple sclerosis: consequences for remyelination and disease progression. Nat Rev Neurol 19, 305-320 (2023). 10.1038/s41582-023-00801-6 37059811

[R90] Liu, X., et al. Small-molecule-induced epigenetic rejuvenation promotes SREBP condensation and overcomes barriers to CNS myelin regeneration. Cell 187, 2465-2484 e2422 (2024). 10.1016/j.cell.2024.04.005 PMC11812128 38701782

